# Neighborhood Qualities Are Related to Better Mental Health, Quality of Life, and Loneliness Over 6 Years: Pathways Through Social Engagement and Social Support to Aging Well

**DOI:** 10.1093/geront/gnaf095

**Published:** 2025-03-06

**Authors:** Christine Stephens, Maryam Bakhshandeh Bavarsad

**Affiliations:** School of Psychology, Massey University, Palmerston North, New Zealand; School of Psychology, Massey University, Palmerston North, New Zealand

**Keywords:** Loneliness, Mental health, Neighborhood qualities, Social engagement, Social support

## Abstract

**Background and Objectives:**

Growing research evidence supports the importance of neighborhoods for the well-being of older people. The aim of this study was to investigate key pathways (social engagement and social support) through which neighborhood qualities (accessibility, safety, and social cohesion) may affect older people’s mental well-being (mental health, quality of life, and loneliness) over 6 years.

**Research Design and Methods:**

A structural equation model was used to test the model while controlling for individual limitations such as physical health and socioeconomic status. The population sample included 2,750 New Zealanders over the age of 55 (*M* = 65.64 ± 6.30) years who responded to Health, Work and Retirement (HWR) longitudinal surveys in 2016 and 2022.

**Results:**

The results showed that greater neighborhood accessibility to important facilities in 2016 predicted better mental health and quality of life and less loneliness in 2022 through provision of social support. Neighborhood social cohesion predicted social engagement, which was related to higher social support predicting better mental health and quality of life and less loneliness in 2022.

**Discussion and Implications:**

These findings confirm a body of research highlighting the importance of neighborhood qualities and show the socially oriented pathways through which neighborhoods support aging well. These findings have direct implications for the development of social policy that focuses on the development of housing situations to support healthy aging.

A growing body of research supports the importance of neighborhoods for the well-being of older people. While older adults’ activities are not limited to the residential neighborhood (Cagney & Cornwell, 2018), it is generally accepted that neighborhoods are an important aspect of daily life (Costa-Font, 2013; Woolrych et al., 2021) and more important to the well-being of older adults than to other age groups (Cagney et al., 2013; Shiovitz-Ezra, 2015). Older people’s perceptions of their neighborhood’s qualities are important indicators of physical and social functioning (Bowling & Stafford, 2007) and life satisfaction (Oswald et al., 2011) and have been shown to predict physical, mental, and social health (Stephens et al., 2019a) over and above expected predictors such as chronic conditions, or socioeconomic status (SES).

Aspects of neighborhoods, including neighborhood design, safety, accessibility to facilities, and neighborhood social cohesion (e.g., Hunter et al., 2011), are important to mental health and quality of life (QoL). Although neighborhood qualities have important implications for policy change, little progress has been made, with many important aspects and moderating or mediating factors remaining understudied (Barnett et al., 2018). The purpose of this article is to further the exploration of neighborhood effects on the well-being of older people by examining pathways between neighborhood qualities and mental well-being. Here, we briefly review the evidence for key neighborhood factors that contribute to specific aspects of mental well-being (mental health, QoL, and loneliness) and suggest conceptual pathways for the mediating roles of social engagement and social support.

## Mental Health, Loneliness, and QoL

Mental health (often assessed as symptoms of depression in this literature) has emerged as an important outcome in neighborhood research which generally shows that perceptions of poor neighborhood quality are related to poorer mental health (Jones-Rounds et al., 2014; Stokes, 2020). A review (Barnett et al., 2018) showed that SES and perceptions of neighborhood trust and safety were most strongly associated with depression symptoms or diagnosis.

Loneliness has emerged as another preventable aspect of older people’s mental well-being. Investigations of contributors to loneliness among older populations in the United States, across Europe, Australia, New Zealand, and Singapore have shown that poorer perceptions of physical and social neighborhood qualities are consistently associated with greater loneliness (Lam, 2022; Massihzadegan & Stokes, 2023; Scharf & de Jong Gierveld, 2008; Shiovitz-Ezra, 2015; Wee et al., 2019) over and above expected predictors such as marital status, gender, and health (Stephens & Phillips, 2022).

QoL is an important aspect of mental well-being that has been rarely examined as an outcome of neighborhood quality. Perceptions of neighborhood social cohesion, safety, and access to important facilities were directly related to QoL in a New Zealand study (Stephens et al., 2019b, 2020). Similarly, Lane et al. (2020), using the same measure of QoL in Singapore, showed direct associations between neighborhood social cohesion and QoL.

## Neighborhood Qualities

Perceived qualities of neighborhoods related to mental well-being include safety, accessibility, and social cohesion. A sense of neighborhood security or safety has been assessed as an important contributor to mental well-being (Barnett et al., 2018; Cain et al., 2018; Vaughan et al., 2016; Yang et al., 2023).

Accessibility to important facilities that support mental well-being and QoL has also been identified as a key neighborhood quality (Stephens et al., 2019b, 2020; Woolrych et al., 2021). Access to shops, services, and transport is positively related to general health (Byles et al., 2014), and a review (Padeiro et al., 2022) showed that access to health care is positively related to mental well-being.

Social cohesion in the neighborhood, conceptualized as norms of trust, solidarity, and reciprocity (Stafford et al., 2003), has emerged as one of the strongest predictors of mental well-being. Perceptions of neighborhood social qualities have been associated positively with loneliness (e.g., Massihzadegan & Stokes, 2023; Shiovitz-Ezra, 2015; Stephens & Phillips, 2022), negatively and causally with depression (Barnett et al., 2018), and negatively with general well-being (Padeiro et al., 2022).

## Theorizing Pathways From Neighborhood Qualities to Mental Well-being

Clarke & Nieuwenhuijsen (2009) critically reviewed the literature to conclude that there was theoretical and empirical neglect of the mechanisms underlying these environment–person relationships. Models to explain the complex interactions of environmental influences have built on theories of person–environment fit which consider how the environment meets the specific needs of older people (e.g., Bigonnesse & Chaudhury, 2020; Wahl et al., 2012) but do not focus on the links to individual mental well-being outcomes. Sociological research has drawn on this gerontological theorizing to focus on the neighborhood social context and well-being of older adults (Cagney & Cornwell, 2018) and there has been consideration of the importance of social space by geographers (Wiles et al., 2009); however, there remains no consensus on how neighborhoods affect individuals (Kaspar et al., 2015). Our theoretical approach is to consider the ways that people’s perceptions of their social and physical environment relate to their social engagement ( Jones-Rounds et al., 2014; Kaspar et al., 2015), which is known to affect mental well-being (Wang et al., 2020). Social engagement has been defined as “the existence and frequency of participation in meaningful activities that involve social interactions among individuals” (Kim & Clarke, 2015, p. 415). It is often used interchangeably with the term “social participation” with similar definitions (Woolrych et al., 2021). Here, we use social engagement as the broader term to encompass both social participation (in terms of contact with other community members) and contributions to community such as volunteering.

### Social Engagement Effects on Aspects of Mental Well-being

Bowling et al. (2007) cited support for the latter section of this proposed pathway in terms of mental health. They reported beneficial effects of social engagement (social contacts and involvement in formal and informal social activities) on mental health. More recently, Zhao and Wu (2022) drew on Activity Theory to explain how engaging socially can prevent loneliness and to demonstrate relationships between social engagement and loneliness among older people. Woolrych et al. (2021) summarized the literature supporting the broader benefits of social engagement including improved QoL and mental well-being, and lower levels of loneliness.

### Social Support as a Mediator

There are also reasons to consider social support as an important mediator of the relationship between social engagement and mental well-being. Social Support has long been accepted as a contributor to well-being (Berkman et al., 2000; Bowling et al., 2007), and Berkman et al. describe conceptual links between social engagement, social support, and mental well-being. Zhao & Wu (2022) recently showed that social support mediated the relationship between social engagement and loneliness, concluding that social participation indirectly reduces loneliness by increasing social support.

### Neighborhood Qualities Affect Social Engagement

There has been limited study of the first step in the proposed pathway, from neighborhood qualities to social engagement, although several authors have considered that the characteristics of places are important for facilitating or impeding social engagement (Cagney et al., 2013; Kaspar et al., 2018). Vaughan et al. (2016) conducted a systematic review and meta-analysis of environmental research to identify six features that significantly impact social participation of older adults. The strongest effects were for neighborliness, living near family and friends (aspects of social cohesion), and civil protection (e.g., safety).

There is limited evidence suggesting that the effects of neighborhood social cohesion on depression may be explained by pathways through social engagement. For example, Choi et al. (2015) showed that, in a U.S. sample, perceptions of neighborhood social cohesion indirectly affected depressive symptoms through frequency of going out. Similarly in China, Wang et al. (2020) found that the negative relationship between social participation and depression was partly mediated by increased contact with neighbors (which suggests stronger neighborhood cohesion). In support of the direction of the effect, Stokes (2020) considered the reverse pathways, finding that although depression was harmful for social integration across mid and older age, its influence was significantly buffered by perceived neighborhood quality.

Other aspects of neighborhood qualities and mental well-being outcomes have been less studied in terms of social engagement. Bowling et al. (2007) suggested that perceptions of poor local facilities for people aged 65 and over were associated with lower social engagement, while Richard et al. (2013) showed that proximity to important facilities (shopping, library, leisure center, physical activity) was the most important contributor to social engagement. Volunteering has also been considered by Dury et al. (2016) who reported that neighborhood connectedness, neighborhood satisfaction, and access to services predicted engagement in voluntary activities in older age. Kim and Clarke (2015) suggested a negative relationship between fear of crime and social participation; however, although sense of neighborhood security or safety is related to mental well-being, little attention has been paid to social engagement as an explanatory pathway.

## The Present Study

The aim of this paper is to test a model in which perceived neighborhood qualities (accessibility, safety, social cohesion) predict levels of social engagement (social participation and volunteering) which predict perceived social support, which in turn, affect older people’s mental health, loneliness, and QoL over time.

Several variables were included to control for their known contribution to explanations of neighborhood qualities, social engagement, social support, and mental well-being. We included age (Oswald et al., 2011), gender, and ethnicity (Lam, 2022; Massihzadegan & Stokes, 2023) to respond to a lack of individual factors accounted for in neighborhood research (Barnett et al., 2018). Social participation has been shown to be particularly beneficial for those living alone and for those experiencing health difficulties (Woolrych et al., 2021); there is strong evidence that SES impacts health (Barnett et al., 2018; Cagney et al., 2013; Cain et al., 2018); tenure insecurity for renters impacts mental well-being in New Zealand (James et al., 2020); and the impacts of physical health must be accounted for (Bowling & Stafford, 2007; Choi et al., 2018; Feng et al., 2018). Māori (the indigenous people of New Zealand) experience higher rates of mental health disorders than the general population and are more likely to live in poorer housing conditions (Bennett & Liu, 2018).

Clarke and Nieuwenhuijsen (2012) and Barnett et al. (2018) note that one main methodological weakness of the studies they reviewed was their cross-sectional nature. We use a prospective design to assess changes in the health outcomes over 6 years (2016–2022). Accordingly, the predictions for the model are:

H-1.1: Perceived Neighborhood Accessibility, Safety, and Social Cohesion in 2016 predict Social Engagement and Social Support in 2016 and Quality of Life, Mental Health, and Loneliness in 2022 (controlling for Physical Health, Living Alone, Ethnicity, SES, Home Ownership, and Quality of Life, Mental Health, and Loneliness in 2016)

H-2.1: The relationship between Perceived Neighborhood Accessibility, Safety, and Social Cohesion in 2016 and Quality of Life, Mental Health, and Loneliness in 2022 will be mediated by Social Engagement and Social Support (2016).

H-2.2: The relationship between Social Engagement (2016) and Quality of Life, Mental Health, and Loneliness in 2022 will be mediated by Social Support (2016).

## Method

### Participants

Data were drawn from responses to the 2016 (T_1_) and 2022 (T_2_) Health, Work and Retirement (HWR) longitudinal surveys (Allen et al., 2019). The HWR is a longitudinal prospective cohort study surveying New Zealanders (randomly selected from the electoral roll) aged 55 and above biennially. Procedures for data collection were approved by the Massey University Human Ethics Committee. Participants for this analysis included respondents who provided complete data on all model variables in the T_1_ and T_2_ survey (*n* = 2,470). Average age of participants was 65.64 ± 6.30 years, and 56.2% (*n* = 1,521) were female, while 26% (*n* = 706) identified as Māori (indigenous people of New Zealand). Descriptive and frequency analysis results are presented in Table 1.

**Table 1.** Characteristics of the Study Population (*n* = 2,705)

Variables	Frequency	Percent
Gender		
Male	1,184	43.80
Female	1,521	56.20
Ethnicity		
New Zealand European	1,769	65.40
Māori	706	26.10
Other	230	8.50
Tenure		
Ownership	2,470	91.30
Nonownership	235	8.70
Living alone		
Yes	496	18.30
No	2,209	81.70

Variables	Mean	*SD*
Age	65.64	6.30
Socioeconomic Status (α = .89)	25.08	5.54
Physical Health (α = .85)	47.86	9.30
Neighborhood Social Cohesion (α = .86)	3.90	0.60
Neighborhood Safety (α = .89)	18.69	2.23
Neighborhood Accessibility (α = .79)	18.38	2.48
Social Participation	1.59	1.30
Volunteering	1.72	1.27
Social Support (α = .86)	79.42	9.54
Loneliness 2016 (α = .71)	1.62	1.63
QOL 2016 (α = .86)	28.95	5.26
Mental Health 2016 (α = .81)	51.23	8.87
Loneliness 2022 (α = .72)	1.41	1.60
QOL 2022 (α = .86)	28.50	5.54
Mental Health 2022 (α = .83)	50.15	9.40

*Notes*: QOL = quality of life; *SD* = standard deviation.

### Measures

Self-reported age, gender, home tenure (owning/renting), and living alone or with others were included as demographic control variables. Ethnicity was assessed as a comparison between non-Māori (0) and Māori (1).

*Physical Health* was assessed using the SF-12v2 Australian and New Zealand form (Ware et al., 2002). Standardized totals were calculated with reference to normative subscale scores and factor coefficients for the New Zealand population (Frieling et al., 2013) relative to a mean score of 50 (*SD* = 10). Higher scores mean better physical health.

*Socioeconomic Status* was assessed using the Economic Living Standards Index-short form (ELSI-SF), a 25-item non-income measure of financial restrictions (Jensen et al., 2005). Scores on the ELSI-SF range from 0 to 31, with higher scores indicating better material living standards. See Table 1 for alpha values in the current sample.

*Neighborhood Social Cohesion* tool (Stafford et al., 2003) measures trust (e.g., “Most people in this area can be trusted”; six items), attachment to neighborhood (e.g., “I really feel part of this area”; four items), practical help (e.g., “I feel comfortable asking my neighbor to lend me $5”; three items), and tolerance or respect (e.g., “People in this area treat each other with respect”; six items) on a 5-point scale anchored at 1 = strongly disagree and 5 = strongly agree. Scores were summed and averaged to provide an index with a range of 1–5. Higher scores on all neighborhood variables mean more positive perceptions of neighborhood qualities.

*Neighborhood Accessibility* was assessed using four items (I can get to the shops easily; I am close to any help I need; I am close enough to important facilities; and I have access to transport) rated on a 5-point scale anchored at 1 = no, definitely not to 5 = yes, definitely. Scores were summed to provide a range of 4–20.

*Neighborhood Safety* was assessed with four items (I feel safe at home; I feel safe in my neighborhood; the neighborhood is peaceful; and I have peace of mind at home), which were rated on a 5-point scale anchored at 1 = no, definitely not to 5 = yes, definitely. Scores were summed to provide a range of 4–20.

*Social Engagement* was assessed in terms of social participation and volunteering. For social participation, participants indicated their membership in seven types of social groups (e.g., sports clubs, community or service organizations that help people). Positive responses to each item were summed providing a total group membership score ranging from 0 to 7. Volunteering was assessed with the mean score of one item: I contribute my time and/or labor to volunteer activities (0–4; never to very often).

*Perceived Social Support* was assessed using the Social Provisions Scale (Cutrona & Russell, 1987). This measure has six subscales that measure separate but highly correlated perceptions of support (Attachment, Social Integration, Reassurance of Worth, Reliable Alliance, Guidance, and Opportunity for Nurturance). Respondents rate the extent to which each of four statements (e.g., “There are people I can depend on to help me if I really need it” or “There is no one I can turn to for guidance in times of stress”) describe how their social relationships are currently supplying each of the provisions using 4-point scales (from completely true to not at all true). The scores are summed (after reversing the negative scores) for each social provision (4–16), and a total social support score is formed by summing the six individual provision scores (24–96). Higher scores are equated to higher social support. Cutrona et al. (1986) demonstrated the validity of its use in predicting the health of older adults. Cutrona and Russell report alpha coefficients for the total scale score from .85 to .92 across a variety of populations.

*Loneliness.* The short-form version of the De Jong Gierveld Loneliness Scale (Gierveld & Van Tilburg, 2006) was administered. The six-item scale includes three items to assess emotional aspects of loneliness (e.g., “I experience a general sense of emptiness”) and three items to assess social (e.g., “There are enough people I feel close to”) aspects of loneliness on a 3-point scale (“no,” “more or less,” and “yes”). Using an item response model scale scores are based on dichotomous item scores with the answer “more or less” always indicating loneliness. Summing the neutral and positive answers (“more or less” and “yes”) on negatively formulated items provides an emotional loneliness score ranging from 0 to 3. Summing neutral and negative answers (“no” and “more or less”) on the positive items provides a social loneliness score ranging from 0 to 3. These scores are summed to provide a total loneliness score of 0–6 with higher scores indicating higher loneliness. Gierveld and Van Tilburg (2010) used large samples to demonstrate the reliability and validity of this version in each of seven countries.

*Quality of Life.* The CASP (Hyde et al., 2003) assesses four conceptual domains of QoL: “control,” “autonomy,” “self-realisation,” and “pleasure.” The CASP-12 requires participants to indicate how often each of 12 statements applies to them (e.g., “I feel left out of things” and “I can do the things that I want to do”) on a 4-point scale ranging from “3” often to “0” never, and summed to provide an overall score with a possible range of 0–36 (36 meaning higher QoL).

*Mental Health* was assessed using the SF-12v2 Australian and New Zealand form (Ware et al., 2002). Standardized totals were calculated with reference to normative subscale scores and factor coefficients for the New Zealand population (Frieling et al., 2013) relative to a mean score of 50 (*SD* = 10). Higher scores mean better mental health.

### Statistical Analysis

Structural Equation Modeling (SEM) was used to assess direct and indirect effects through AMOS software version 29 using the Maximum Likelihood Estimation Method. Missing data were addressed through regression imputation using AMOS software (Baraldi & Enders, 2010).

Absolute Fit Indices (Goodness-of-Fit Index [GFI], Adjusted GFI [AGFI], and Root Mean Square Error of Approximation [RMSEA]), Comparative Fit Indices (Comparative Fit Index [CFI], and Normed Fit Index [NFI]), and Residual-Based Fit Indices (Standardized Root Mean Square Residual [SRMR]) were considered to evaluate the model’s goodness of fit. Criteria for an appropriate fit were: GFI, AGFI, and CFI values higher than 0.90 (Kline, 1998) and RMSEA and SRMR lower than 0.08 (Schreiber et al., 2006).

Significance of indirect effects was assessed using standard error and 95% confidence intervals (CIs) calculated from 5,000 resamples generated through a bias-corrected bootstrapping method.

## Results

**Figure 1.**
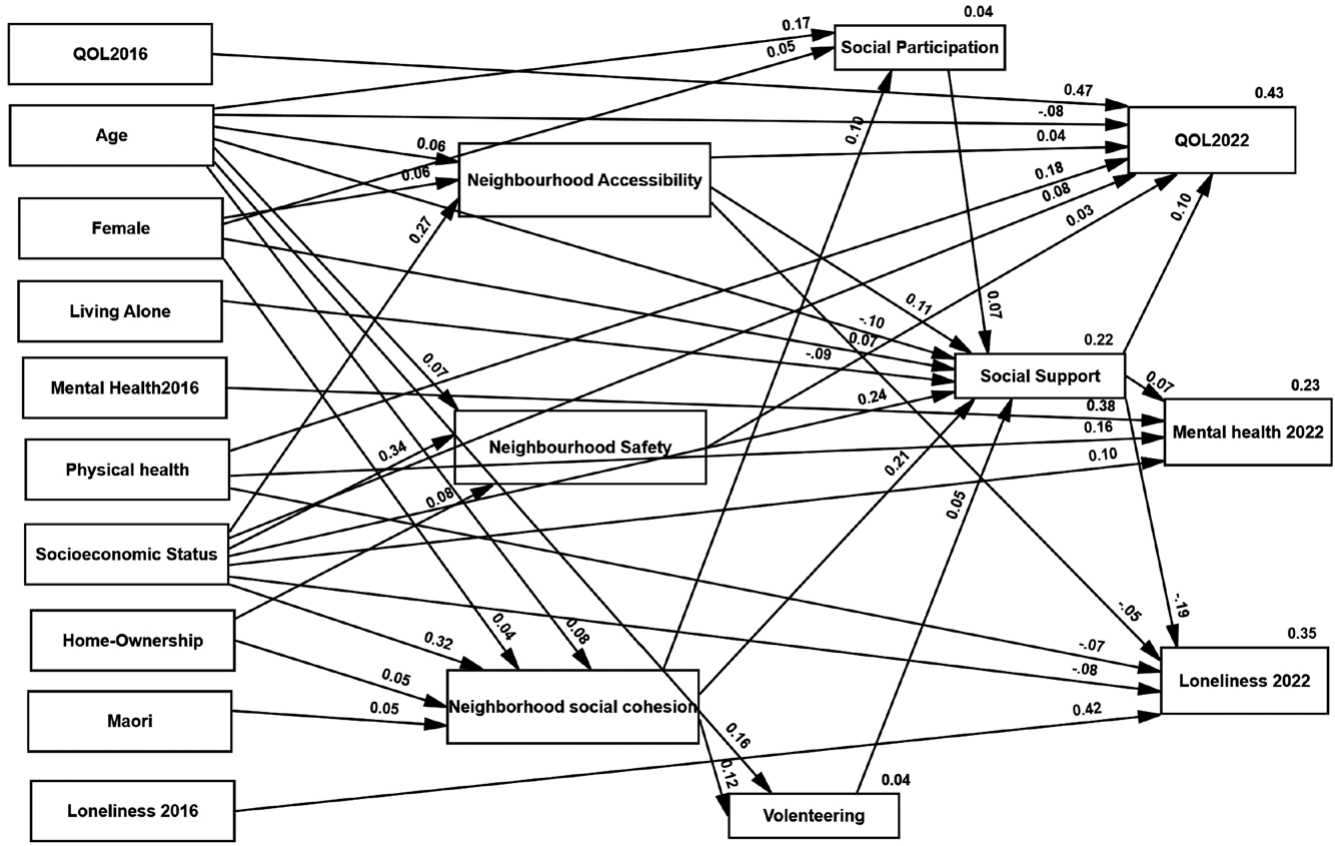
Results of the SEM model showing significant direct beta values between pathway variables. SEM = Structural Equation Modeling.

The theoretical model was modified by deleting insignificant paths and modification indices. The final model (Figure 1) demonstrated a good fit (GFI = 0.95; AGFI = 0.91; NFI = 0.91; CFI = 0.92; RMSEA = 0.07; SRMR = 0.07). The model explained 43%, 35%, and 23% of the variance in QoL, Loneliness, and Mental Health, respectively. The standardized path coefficients and *p*-values for the SEM are presented in Table 2.

**Table 2.** The Direct Relationships Between Dependent and Independent Variables in the Structural Equation Model

Variables	*B*	*SE*	CR	*p*	β
QOL 2022					
Social Support	0.06	0.01	6.45	^***^	0.10
Age	−0.06	0.01	−6.04	^***^	−0.08
Neighborhood Safety	0.08	0.03	2.28	.02	0.03
Socioeconomic Status	0.08	0.02	4.69	^***^	0.09
QOL 2016	0.48	0.02	30.89	^***^	0.47
Physical Health	0.10	0.01	11.28	^***^	0.18
Neighborhood Accessibility	0.08	0.03	2.69	.01	0.04
Loneliness 2022					
Neighborhood Accessibility	−0.03	0.01	−3.31	^***^	−0.05
Social Support	−0.03	0.00	−10.55	^***^	−0.19
Socioeconomic Status	−0.02	0.01	−4.52	^***^	−0.08
Physical Health	−0.01	0.00	−4.12	^***^	−0.07
Loneliness 2016	0.42	0.02	26.10	^***^	0.42
Mental Health 2022					
Mental Health 2016	0.38	0.02	23.45	^***^	0.38
Social Support	0.07	0.02	3.87	^***^	0.07
Physical Health	0.16	0.02	9.11	^***^	0.16
Socioeconomic Status	0.17	0.03	4.97	^***^	0.10
Social Cohesion					
Home Ownership	0.11	0.04	2.80	.01	0.05
Socioeconomic Status	0.04	0.00	17.30	^***^	0.32
Female	0.05	0.02	2.70	.01	0.04
Age	0.01	0.00	4.42	^***^	0.08
Māori	0.07	0.02	3.23	.00	0.05
Social Support					
Living alone	−2.19	0.38	−5.73	^***^	−0.09
Volunteering	0.38	0.13	2.87	.00	0.05
Social Cohesion	3.33	0.27	12.40	^***^	0.21
Social Participation	0.47	0.13	3.67	^***^	0.07
Neighborhood Accessibility	0.42	0.06	6.72	^***^	0.11
Female	1.40	0.30	4.74	^***^	0.08
Socioeconomic Status	0.42	0.03	13.19	^***^	0.24
Age	−0.15	0.02	−6.39	^***^	−0.10
Neighborhood Accessibility					
Female	0.31	0.09	3.59	^***^	0.06
Socioeconomic Status	0.13	0.01	14.89	^***^	0.27
Age	0.03	0.01	3.50	^***^	0.06
Neighborhood Safety					
Home Ownership	0.64	0.14	4.72	^***^	0.08
Socioeconomic Status	0.14	0.01	18.73	^***^	0.34
Age	0.02	0.01	3.88	^***^	0.07
Social Participation					
Social Cohesion	0.22	0.04	5.40	^***^	0.10
Female	0.12	0.04	2.73	.01	0.05
Age	0.04	0.00	8.93	^***^	0.17
Volunteering					
Social Cohesion	0.26	0.04	6.34	^***^	0.12
Age	0.03	0.00	8.42	^***^	0.16

*Notes*: QOL = quality of life; *SE* = standard error; CR = critical ration.

^***^ *p* < .001.

H-1.1 was partially supported. Among the Neighborhood variables, only Social Cohesion had a positive direct effect on Social Participation and Volunteering (β = 0.10, *p* < .001 and β = 0.12, *p* < .001, respectively). Neighborhood Accessibility and Social Cohesion had significant direct effects on Social Support (β = 0.11, *p* < .001 and β = 0.21, *p* < .001, respectively). Neighborhood Safety directly affected only Quality of Life (β = 0.03, *p* < .05).

H-2.1 was partially supported. Neighborhood Accessibility was directly (β = 0.04, *p* < .05), and indirectly, through Social Support, positively related to QoL (β = 0.02; 95% CI [0.01–0.04], *p* < .001). Neighborhood Accessibility exhibited a negative direct relationship with Loneliness (β = −0.05, *p* < .001) and a negative indirect association through Social Support (β = −0.01; 95% CI [−0.02 to −0.01], *p* < .001). Neighborhood Accessibility was only related to Mental Health indirectly through Social Support (β = 0.03; 95% CI [0.01–0.05], *p* < .001). Higher Neighborhood Accessibility was related to higher perceptions of Social Support, which in turn were related to better Mental Health.

There was no direct effect between Neighborhood Social Cohesion and QOL, Loneliness, or Mental Health. However, there were significant sequential indirect paths from Social Cohesion to Social Participation to Social Support to QOL (β = 0.01; 95% CI [0.00–0.01], *p* < .001), Loneliness (β = −0.003; 95% CI [−0.006 to −0.001], *p* < .01), and Mental Health (β = 0.007; 95% CI [0.002–0.014], *p* < .01). Greater perceptions of Neighborhood Social Cohesion were related to higher levels of Social Participation, leading to lower levels of Loneliness and hence to better Mental Health. The same pathways were seen for Volunteering, revealing significant sequential indirect paths from higher Social Cohesion to more Volunteering to higher Social Support to greater QOL (β = 0.006; 95% CI [0.001–0.011], *p* < .01), less Loneliness (β = −0.003; 95% CI [−0.006 to −0.001], *p* < .01), and better Mental Health (β = 0.007; 95% CI [0.002–0.014], *p* < .01) (Table 3).

**Table 3.** Mediation Effects of Social Support, Social Participation, and Volunteering on Quality of Life, Mental Health, and Loneliness

Regression path				β	95% CI		*p*
					Lower	Upper	
Neighborhood Accessibility		Social Support	QOL	0.02	0.01	0.04	^***^
Neighborhood Accessibility		Social Support	Mental Health	0.03	0.01	0.05	^**^
Neighborhood Accessibility		Social Support	Loneliness	−0.01	−0.02	−0.01	^***^
Social Cohesion		Social Support	QOL	0.19	0.12	0.28	^***^
Social Cohesion		Social Support	Mental Health	0.23	0.10	0.37	^**^
Social Cohesion		Social Support	Loneliness	−0.10	−0.13	−0.08	^***^
Social Participation		Social Support	QOL	0.03	0.01	0.05	^**^
Social Participation		Social Support	Mental Health	0.03	0.01	0.06	^**^
Social Participation		Social Support	Loneliness	−0.01	−0.02	−0.01	^**^
Volunteering		Social Support	QOL	0.02	0.01	0.04	^**^
Volunteering		Social Support	Mental Health	0.03	0.01	0.05	^**^
Volunteering		Social Support	Loneliness	−0.01	−0.02	0.00	^**^
Social Cohesion		Social Participation	Social Support	0.11	0.04	0.18	^**^
Social Cohesion		Volunteering	Social Support	0.10	0.03	0.18	^**^
Social Cohesion	Social Participation	Social Support	QOL	0.01	0.00	0.01	^**^
Social Cohesion	Social Participation	Social Support	Mental Health	0.01	0.00	0.01	^**^
Social Cohesion	Social Participation	Social Support	Loneliness	0.00	−0.01	0.00	^**^
Social Cohesion	Volunteering	Social Support	QOL	0.01	0.00	0.01	^**^
Social Cohesion	Volunteering	Social Support	Mental Health	0.01	0.00	0.01	^**^
Social Cohesion	Volunteering	Social Support	Loneliness	0.00	−0.01	0.00	^**^

*Notes*: CI = confidence interval; QOL = quality of life.

^**^ *p* < .01.

^***^ *p* < .001.

H-2.2 was supported. Both Social Participation and Volunteering had a significant positive effect on Social Support (β = 0.07, *p* < .001 and β = 0.05, *p* < .01, respectively). Although there was no significant direct effect between Social Participation and Volunteering and the outcome variables, there was a positive indirect effect between these variables and outcomes through Social Support.

## Discussion

These findings confirm the importance of neighborhoods to older adults’ mental well-being, over and above physical health and other important individual limitations. Two important aspects of neighborhood conditions and their impacts on subsequent mental well-being were highlighted by the analysis: neighborhood social cohesion and access to facilities were related to changes in mental health, QoL, and loneliness, supporting findings highlighted by Barnett et al. (2018). Perceptions of neighborhood safety were directly related only to QoL. The final model demonstrates pathways from social cohesion and neighborhood accessibility to changes in mental health over six years.

Neighborhood accessibility was directly related to QoL and loneliness (cf. Byles et al., 2014; Padeiro et al., 2022; Stephens et al., 2019b, 2020; Woolrych et al., 2021). The indirect route highlighted by the present findings was through social support to mental health, QoL, and loneliness. Neighborhoods that provide opportunities to access the facilities valued by older people are related to higher feelings of social support and subsequent positive mental well-being. There has been little focused research on perceptions of social support found in neighborhoods, and much of that which has included social support is related to social participation (e.g., Bowling et al., 2007; Levasseur et al., 2015). Berkman et al. (2000) suggest that frequency of contact with social networks enhances perceptions of social support; neighborhood facilities such as shops and libraries which provide these opportunities for contact may directly enhance perceptions of social support although they are not seen as formal social participation (such as belonging to societies or churches). Further investigation of the ways in which social support is provided by neighborhoods would be fruitful.

Social cohesion in the neighborhood was supported as an important predictor of mental health (cf. Cain et al., 2017; Choi et al., 2023; Greenfield & Reyes, 2015; Honjo et al., 2018; Vaughan et al., 2016), QoL (cf. Padeiro et al., 2022; Stephens et al., 2020), and loneliness (cf. Massihzadegan & Stokes, 2023; Shiovitz-Ezra, 2015; Stephens & Phillips, 2022). The important contribution of the present findings was clarifying the pathways to these positive outcomes which were not direct. Neighborhood social cohesion was related to both social participation and volunteering, as forms of social engagement. Social engagement, in turn, was related to social support which predicted mental health, QoL, and less loneliness 6 years later. A socially cohesive neighborhood high in trust of neighbors facilitates social engagement with positive outcomes including higher perceived social support and subsequent higher levels of mental health, QoL, and lower loneliness.

Contrary to our hypotheses, social engagement (social participation and volunteering) was only facilitated by perceptions of higher neighborhood social cohesion. A recent review (Townsend et al., 2021) concluded that neighborhood cohesion plays a vital role in maintaining quality social participation. Reviewed findings also show that both individual and community-level changes can improve social participation, and our findings support this suggestion. After age, social cohesion had the strongest relationship to social participation, supporting the importance of both individually appropriate and community-level interventions to promote social cohesion and enhance social engagement among older people.

Social support was a lynchpin in the final model. Although social support has proved to be an important contributor to health (Berkman et al., 2000), it has been largely neglected in environmental gerontology, although Zhao and Wu (2022) recently highlighted the mediating role of social support in the relationship between social participation and loneliness. In the current findings, both neighborhood accessibility and social cohesion were related to social support which in turn affected all aspects of mental well-being. Among the hypothesized variables, social support had the strongest relationships with mental health, QoL, and loneliness. It is clear that perceptions of social support can be supported by good neighborhood qualities which should be encouraged. However, there remains work to be done. Social support has many facets, and different types of social support are provided by different aspects of social life (Cutrona, 1986). More complex analyses of the types of support provided by different aspects of neighborhood life and more in-depth exploration of the provision of support will be useful in this context.

It is also important to recognize the importance of the demographic variables when considering the present findings. SES contributed directly (and often most strongly) to all neighborhood variables, to social support, and to all aspects of mental well-being. This is not a surprising finding, given our recognition of the links between inequalities and health and must not be forgotten when considering interventions to improve health. Age and gender also explained variance in some neighborhood perceptions and health outcomes, and such individual differences in needs should also be considered when thinking about the types of neighborhood facilities provided. For instance, ethnic differences showed that Māori report a slightly higher level of neighborhood social cohesion compared to non-Māori, and this accords with the importance of social cohesion in terms of connections between individuals, whanau (wider family), and land or place in Māori culture (Reid et al., 2016; Spoonley et al., 2020). This sense of social cohesion is linked to mental well-being through increased social engagement and social support for all; however, these results emphasize that the links between individual or group needs and the provision of supportive neighborhoods as part of the pathway must be recognized. Woolrych et al. (2021) describe how social participation in older age involves interrelated physical, psychological, and social processes that operate across a range of environmental settings. Older people are not one homogenous group, and it is important to include the recognition of individual differences such as age and gender and broader social differences such as ethnicity, and SES in the development of supportive neighborhood environments.

The important implication of this body of work is for social policy. Housing and neighborhoods have been shown to have important impacts on the well-being of older people which are amenable to change through government policy (Hunter et al., 2011). Neighborhoods remain a very practical and achievable focus for government policy to improve the health of older people in an aging society. These findings provide a framework within which to focus research with particular communities about their needs for neighborhood improvement, particularly in deprived or neglected areas.

## Data Availability

Data used in this study are available on request. Details are found on the Health and Ageing Research Team web site at: www.massey.ac.nz/hwr-data. This observational study was not preregistered.
